# Poly[bis­(*N*,*N*-dimethyl­formamide)(μ-formato)(μ_5_-4-oxidoisophthalato)dizinc(II)]

**DOI:** 10.1107/S1600536809028566

**Published:** 2009-07-25

**Authors:** Young Ho Jhon, Jaheon Kim

**Affiliations:** a Department of Chemistry, and CAMDRC, Soongsil University, 511 Sangdo-dong, Dongjak-Ku, Seoul 156-743, Republic of Korea

## Abstract

The title compound, [Zn_2_(CHO_2_)(C_8_H_3_O_5_)(C_3_H_7_NO)_2_]_*n*_, is a three-dimensional metal–organic framework, of which two independent Zn^II^ atoms (denoted Zn1 and Zn2) are linked by both 4-oxidoisophthalate and formate bridging ligands. The 4-oxidoisophthalate ligands link two Zn1-type and three Zn2-type atoms, forming a corrugated sheet roughly parallel to the *ac* plane. The formate ions join two neighboring sheets along the *b* axis, forming a three-dimensional network. Two independent dimethylformamide ligands are coordinated to separate Zn^II^ atoms and fill the voids provided by the framework. Both types of Zn^II^ atoms have a distorted trigonal-bipyramidal coordination geometry.

## Related literature

Zn ions and 4-hydroxy­isophthalates can be assembled in a different way due to an auxiliary pyridyl ligand; see: Zhang *et al.* (2004 ▶).
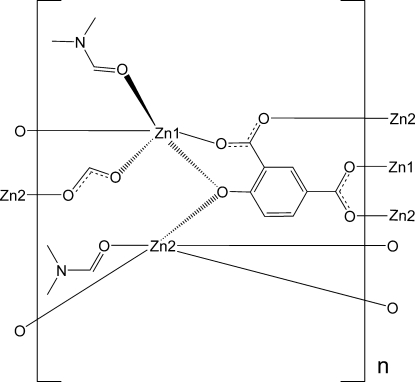


## Experimental

### 

#### Crystal data

[Zn_2_(CHO_2_)(C_8_H_3_O_5_)(C_3_H_7_NO)_2_]
*M* *_r_* = 501.05
Monoclinic, 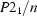

*a* = 9.1190 (4) Å
*b* = 14.7355 (6) Å
*c* = 14.4711 (6) Å
β = 107.752 (1)°
*V* = 1851.94 (13) Å^3^
*Z* = 4
Mo *K*α radiation
μ = 2.64 mm^−1^
*T* = 173 K
0.25 × 0.20 × 0.10 mm

#### Data collection

Bruker SMART CCD diffractometer
Absorption correction: multi-scan (*SADABS*; Sheldrick, 1996 ▶) *T*_min_ = 0.551, *T*_max_ = 0.768
9831 measured reflections
3343 independent reflections
2881 reflections with *I* > 2σ(*I*)
*R*_int_ = 0.066

#### Refinement

*R*[*F* ^2^ > 2σ(*F* ^2^)] = 0.042
*wR*(*F* ^2^) = 0.097
*S* = 1.18
3343 reflections
257 parameters
H-atom parameters constrained
Δρ_max_ = 0.76 e Å^−3^
Δρ_min_ = −0.99 e Å^−3^

### 

Data collection: *SMART* (Bruker, 1997 ▶); cell refinement: *SAINT* (Bruker, 1997 ▶); data reduction: *SAINT*; program(s) used to solve structure: *SHELXS97* (Sheldrick, 2008 ▶); program(s) used to refine structure: *SHELXL97* (Sheldrick, 2008 ▶); molecular graphics: *SHELXTL* (Sheldrick, 2008 ▶); software used to prepare material for publication: *SHELXTL* and *MS Modeling* (Accelrys, 2005 ▶).

## Supplementary Material

Crystal structure: contains datablocks I, global. DOI: 10.1107/S1600536809028566/xu2557sup1.cif

Structure factors: contains datablocks I. DOI: 10.1107/S1600536809028566/xu2557Isup2.hkl

Additional supplementary materials:  crystallographic information; 3D view; checkCIF report

## Figures and Tables

**Table 1:** Selected bond lengths (Å)

Zn1—O4^i^	1.960 (3)
Zn1—O2	1.972 (3)
Zn1—O6	1.977 (3)
Zn1—O1	2.083 (2)
Zn1—O8	2.127 (3)
Zn2—O5^i^	1.978 (3)
Zn2—O1	2.029 (3)
Zn2—O3^ii^	2.038 (3)
Zn2—O7^iii^	2.075 (3)
Zn2—O9	2.114 (3)

Symmetry codes: (i) 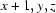; (ii) 
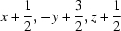
; (iii) 
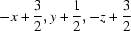
.

**Fig. 1.**
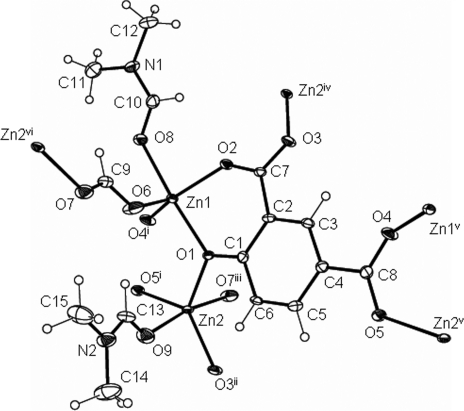
The fragment structure of (I) is shown with the atomic numbering scheme. Displacement ellipsoids are drawn at the 50% probability level and H atoms are shown as small spheres of arbitrary radii. [Symmetry codes: (i) x + 1, y, z; (ii) x + 1/2, -y + 3/2, z + 1/2; (iii) -x + 3/2, y + 1/2, -z + 3/2; (iv) x - 1/2, -y + 3/2, z - 1/2; (v) x - 1, y, z; (vi) -x + 3/2, y - 1/2, -z + 3/2]

## supplementary crystallographic information

### Experimental

The hydroxybenzene-2,4-dicarboxylic acid was purchased from TCI. Hydroxybenzene-2,4-dicarboxylic acid (10 mg, 0.06 mmol) and Zn(NO_3_)_2_.6H_2_O (32 mg, 0.11 mmol) were dissolved in the mixture of *N,N'*-dimethylformamide (1.0 ml) and H_2_O (0.05 ml) solution in 20 ml vial. Then the vial was capped tightly, and placed at 105 °C for 7 days to obtain the crystals for the X-ray crystallographic study.

### Refinement

Hydrogen atoms were placed at calculated positions (C—H = 0.95 or 0.98 Å) and were treated as riding on their attached C atoms with U(H) set to 1.2 times *U*_eq_(C).

### Crystal data

**Table:**

[Zn_2_(CHO_2_)(C_8_H_3_O_5_)(C_3_H_7_NO)_2_]	*F*(000) = 1016
*M**_r_* = 501.05	*D*_x_ = 1.797 Mg m^−^^3^
Monoclinic, *P*2_1_/*n*	Mo *K*α radiation, λ = 0.71073 Å
Hall symbol: -P 2yn	Cell parameters from 5137 reflections
*a* = 9.1190 (4) Å	θ = 2.4–28.3°
*b* = 14.7355 (6) Å	µ = 2.64 mm^−^^1^
*c* = 14.4711 (6) Å	*T* = 173 K
β = 107.752 (1)°	Rectangular, light yellow
*V* = 1851.94 (13) Å^3^	0.25 × 0.20 × 0.10 mm
*Z* = 4	

### Data collection

**Table:**

Bruker SMART CCD diffractometer	3343 independent reflections
Radiation source: fine-focus sealed tube	2881 reflections with *I* > 2σ(*I*)
graphite	*R*_int_ = 0.066
φ and ω scans	θ_max_ = 25.2°, θ_min_ = 2.0°
Absorption correction: multi-scan (*SADABS*; Sheldrick, 1996)	*h* = −10→10
*T*_min_ = 0.551, *T*_max_ = 0.768	*k* = −17→13
9831 measured reflections	*l* = −16→17

### Refinement

**Table:**

Refinement on *F*^2^	Primary atom site location: structure-invariant direct methods
Least-squares matrix: full	Secondary atom site location: difference Fourier map
*R*[*F*^2^ > 2σ(*F*^2^)] = 0.042	Hydrogen site location: inferred from neighbouring sites
*wR*(*F*^2^) = 0.097	H-atom parameters constrained
*S* = 1.18	*w* = 1/[σ^2^(*F*_o_^2^) + (0.0404*P*)^2^ + 1.5088*P*] where *P* = (*F*_o_^2^ + 2*F*_c_^2^)/3
3343 reflections	(Δ/σ)_max_ = 0.011
257 parameters	Δρ_max_ = 0.76 e Å^−^^3^
0 restraints	Δρ_min_ = −0.99 e Å^−^^3^

### Special details

**Table:**

Geometry. All e.s.d.'s (except the e.s.d. in the dihedral angle between two l.s. planes) are estimated using the full covariance matrix. The cell e.s.d.'s are taken into account individually in the estimation of e.s.d.'s in distances, angles and torsion angles; correlations between e.s.d.'s in cell parameters are only used when they are defined by crystal symmetry. An approximate (isotropic) treatment of cell e.s.d.'s is used for estimating e.s.d.'s involving l.s. planes.
Refinement. Refinement of *F*^2^ against ALL reflections. The weighted *R*-factor *wR* and goodness of fit *S* are based on *F*^2^, conventional *R*-factors *R* are based on *F*, with *F* set to zero for negative *F*^2^. The threshold expression of *F*^2^ > σ(*F*^2^) is used only for calculating *R*-factors(gt) *etc*. and is not relevant to the choice of reflections for refinement. *R*-factors based on *F*^2^ are statistically about twice as large as those based on *F*, and *R*- factors based on ALL data will be even larger.

### Fractional atomic coordinates and isotropic or equivalent isotropic displacement parameters (Å^2^)

**Table:**

	*x*	*y*	*z*	*U*_iso_*/*U*_eq_	
Zn1	0.72276 (5)	0.65094 (3)	0.63563 (3)	0.01168 (14)	
Zn2	0.81689 (5)	0.74739 (3)	0.86581 (3)	0.01100 (14)	
O1	0.6482 (3)	0.70324 (18)	0.74736 (18)	0.0122 (6)	
O2	0.5239 (3)	0.69042 (19)	0.54535 (18)	0.0154 (6)	
O3	0.2738 (3)	0.7065 (2)	0.48774 (18)	0.0170 (6)	
O4	−0.0743 (3)	0.7091 (2)	0.67563 (19)	0.0205 (7)	
O5	0.0125 (3)	0.7240 (2)	0.83755 (19)	0.0178 (6)	
O6	0.7196 (4)	0.5270 (2)	0.6874 (2)	0.0311 (8)	
O7	0.7029 (4)	0.3780 (2)	0.6904 (2)	0.0240 (7)	
O8	0.7888 (3)	0.6054 (2)	0.51459 (19)	0.0209 (7)	
O9	0.8384 (4)	0.6248 (2)	0.9446 (2)	0.0255 (7)	
N1	0.7454 (4)	0.5608 (2)	0.3589 (2)	0.0209 (8)	
N2	0.7907 (5)	0.4794 (3)	0.9734 (3)	0.0256 (9)	
C1	0.5022 (4)	0.7046 (3)	0.7454 (3)	0.0114 (8)	
C2	0.3757 (4)	0.7015 (3)	0.6584 (3)	0.0110 (8)	
C3	0.2254 (4)	0.7048 (3)	0.6639 (3)	0.0123 (8)	
H3	0.1419	0.7020	0.6055	0.015*	
C4	0.1940 (4)	0.7121 (3)	0.7516 (3)	0.0129 (8)	
C5	0.3178 (4)	0.7163 (3)	0.8370 (3)	0.0143 (8)	
H5	0.2987	0.7221	0.8976	0.017*	
C6	0.4675 (4)	0.7120 (3)	0.8342 (3)	0.0145 (8)	
H6	0.5495	0.7139	0.8933	0.017*	
C7	0.3937 (4)	0.6988 (3)	0.5587 (3)	0.0108 (8)	
C8	0.0325 (4)	0.7156 (3)	0.7551 (3)	0.0130 (8)	
C9	0.7218 (4)	0.4501 (3)	0.6507 (3)	0.0163 (9)	
H9	0.7389	0.4467	0.5893	0.020*	
C10	0.7002 (5)	0.5902 (3)	0.4312 (3)	0.0178 (9)	
H10	0.5933	0.6007	0.4196	0.021*	
C11	0.9072 (5)	0.5454 (3)	0.3685 (3)	0.0322 (12)	
H11A	0.9713	0.5693	0.4310	0.039*	
H11B	0.9334	0.5765	0.3157	0.039*	
H11C	0.9258	0.4802	0.3652	0.039*	
C12	0.6343 (6)	0.5446 (3)	0.2635 (3)	0.0292 (11)	
H12A	0.5303	0.5583	0.2659	0.035*	
H12B	0.6395	0.4809	0.2453	0.035*	
H12C	0.6584	0.5838	0.2154	0.035*	
C13	0.8027 (5)	0.5464 (3)	0.9161 (3)	0.0238 (10)	
H13	0.7829	0.5339	0.8490	0.029*	
C14	0.8165 (8)	0.4940 (4)	1.0762 (4)	0.0464 (15)	
H14A	0.8540	0.5560	1.0933	0.056*	
H14B	0.8933	0.4506	1.1134	0.056*	
H14C	0.7197	0.4853	1.0912	0.056*	
C15	0.7510 (7)	0.3869 (3)	0.9373 (4)	0.0399 (13)	
H15A	0.7490	0.3835	0.8693	0.048*	
H15B	0.6493	0.3709	0.9422	0.048*	
H15C	0.8280	0.3444	0.9761	0.048*	

### Atomic displacement parameters (Å^2^)

**Table:**

	*U*^11^	*U*^22^	*U*^33^	*U*^12^	*U*^13^	*U*^23^
Zn1	0.0099 (2)	0.0170 (3)	0.0074 (2)	0.00076 (18)	0.00155 (16)	−0.00134 (18)
Zn2	0.0099 (2)	0.0173 (3)	0.0057 (2)	−0.00064 (17)	0.00219 (16)	−0.00158 (17)
O1	0.0084 (13)	0.0204 (16)	0.0076 (13)	0.0010 (11)	0.0020 (10)	−0.0036 (11)
O2	0.0117 (14)	0.0273 (17)	0.0078 (13)	0.0023 (11)	0.0038 (11)	0.0010 (12)
O3	0.0114 (14)	0.0310 (18)	0.0074 (13)	0.0011 (12)	0.0012 (11)	0.0015 (12)
O4	0.0092 (14)	0.040 (2)	0.0103 (14)	−0.0031 (12)	0.0003 (11)	−0.0018 (13)
O5	0.0139 (14)	0.0320 (18)	0.0095 (14)	0.0036 (12)	0.0066 (11)	0.0023 (12)
O6	0.064 (2)	0.0158 (18)	0.0153 (16)	0.0015 (15)	0.0140 (16)	−0.0019 (13)
O7	0.0392 (19)	0.0185 (17)	0.0145 (15)	0.0019 (13)	0.0087 (13)	0.0021 (12)
O8	0.0177 (15)	0.0326 (19)	0.0118 (14)	0.0054 (13)	0.0038 (12)	−0.0032 (13)
O9	0.0361 (19)	0.0203 (18)	0.0192 (16)	0.0002 (14)	0.0069 (14)	0.0039 (13)
N1	0.028 (2)	0.022 (2)	0.0132 (17)	0.0032 (15)	0.0076 (15)	−0.0039 (15)
N2	0.043 (2)	0.018 (2)	0.0175 (19)	0.0009 (17)	0.0119 (17)	0.0012 (15)
C1	0.0093 (19)	0.012 (2)	0.0129 (19)	−0.0008 (15)	0.0030 (15)	−0.0017 (15)
C2	0.016 (2)	0.011 (2)	0.0060 (18)	−0.0021 (15)	0.0038 (15)	−0.0003 (15)
C3	0.0117 (19)	0.016 (2)	0.0080 (18)	−0.0010 (15)	0.0008 (15)	−0.0016 (15)
C4	0.013 (2)	0.016 (2)	0.0106 (19)	0.0001 (15)	0.0047 (15)	0.0002 (15)
C5	0.016 (2)	0.018 (2)	0.0096 (19)	−0.0024 (16)	0.0051 (16)	−0.0021 (16)
C6	0.0105 (19)	0.024 (2)	0.0062 (18)	0.0013 (16)	−0.0011 (15)	−0.0032 (16)
C7	0.014 (2)	0.011 (2)	0.0069 (18)	0.0002 (15)	0.0035 (15)	0.0006 (15)
C8	0.012 (2)	0.015 (2)	0.013 (2)	0.0000 (15)	0.0051 (16)	0.0011 (15)
C9	0.018 (2)	0.020 (2)	0.0104 (19)	−0.0015 (17)	0.0038 (16)	−0.0009 (17)
C10	0.018 (2)	0.020 (2)	0.017 (2)	0.0017 (17)	0.0082 (17)	0.0006 (17)
C11	0.031 (3)	0.044 (3)	0.025 (2)	0.009 (2)	0.014 (2)	−0.004 (2)
C12	0.037 (3)	0.037 (3)	0.015 (2)	0.002 (2)	0.008 (2)	−0.008 (2)
C13	0.030 (3)	0.031 (3)	0.009 (2)	0.002 (2)	0.0045 (18)	0.0003 (19)
C14	0.095 (5)	0.026 (3)	0.026 (3)	0.000 (3)	0.031 (3)	0.003 (2)
C15	0.065 (4)	0.024 (3)	0.024 (3)	−0.008 (2)	0.003 (2)	0.004 (2)

### Geometric parameters (Å, °)

**Table:**

Zn1—O4^i^	1.960 (3)	N2—C15	1.465 (6)
Zn1—O2	1.972 (3)	C1—C6	1.418 (5)
Zn1—O6	1.977 (3)	C1—C2	1.425 (5)
Zn1—O1	2.083 (2)	C2—C3	1.397 (5)
Zn1—O8	2.127 (3)	C2—C7	1.502 (5)
Zn2—O5^i^	1.978 (3)	C3—C4	1.389 (5)
Zn2—O1	2.029 (3)	C3—H3	0.9500
Zn2—O3^ii^	2.038 (3)	C4—C5	1.398 (5)
Zn2—O7^iii^	2.075 (3)	C4—C8	1.489 (5)
Zn2—O9	2.114 (3)	C5—C6	1.379 (5)
O1—C1	1.323 (4)	C5—H5	0.9500
O2—C7	1.266 (4)	C6—H6	0.9500
O3—C7	1.256 (4)	C9—H9	0.9500
O3—Zn2^iv^	2.038 (3)	C10—H10	0.9500
O4—C8	1.264 (5)	C11—H11A	0.9800
O4—Zn1^v^	1.960 (3)	C11—H11B	0.9800
O5—C8	1.267 (4)	C11—H11C	0.9800
O5—Zn2^v^	1.978 (3)	C12—H12A	0.9800
O6—C9	1.255 (5)	C12—H12B	0.9800
O7—C9	1.244 (5)	C12—H12C	0.9800
O7—Zn2^vi^	2.075 (3)	C13—H13	0.9500
O8—C10	1.250 (5)	C14—H14A	0.9800
O9—C13	1.236 (5)	C14—H14B	0.9800
N1—C10	1.311 (5)	C14—H14C	0.9800
N1—C11	1.457 (6)	C15—H15A	0.9800
N1—C12	1.462 (6)	C15—H15B	0.9800
N2—C13	1.316 (6)	C15—H15C	0.9800
N2—C14	1.448 (6)		
			
O4^i^—Zn1—O2	131.40 (12)	C3—C4—C8	121.0 (3)
O4^i^—Zn1—O6	114.44 (14)	C5—C4—C8	120.6 (3)
O2—Zn1—O6	114.00 (13)	C6—C5—C4	120.8 (3)
O4^i^—Zn1—O1	96.38 (11)	C6—C5—H5	119.6
O2—Zn1—O1	87.33 (10)	C4—C5—H5	119.6
O6—Zn1—O1	90.09 (12)	C5—C6—C1	121.8 (3)
O4^i^—Zn1—O8	84.39 (11)	C5—C6—H6	119.1
O2—Zn1—O8	88.94 (10)	C1—C6—H6	119.1
O6—Zn1—O8	93.51 (12)	O3—C7—O2	120.5 (3)
O1—Zn1—O8	175.63 (11)	O3—C7—C2	117.4 (3)
O5^i^—Zn2—O1	105.56 (11)	O2—C7—C2	122.1 (3)
O5^i^—Zn2—O3^ii^	131.40 (11)	O4—C8—O5	124.9 (3)
O1—Zn2—O3^ii^	122.80 (10)	O4—C8—C4	117.5 (3)
O5^i^—Zn2—O7^iii^	93.28 (12)	O5—C8—C4	117.6 (3)
O1—Zn2—O7^iii^	90.76 (11)	O7—C9—O6	123.7 (4)
O3^ii^—Zn2—O7^iii^	90.77 (11)	O7—C9—H9	118.2
O5^i^—Zn2—O9	91.22 (12)	O6—C9—H9	118.2
O1—Zn2—O9	95.94 (12)	O8—C10—N1	124.1 (4)
O3^ii^—Zn2—O9	80.11 (12)	O8—C10—H10	117.9
O7^iii^—Zn2—O9	170.64 (11)	N1—C10—H10	117.9
C1—O1—Zn2	120.8 (2)	N1—C11—H11A	109.5
C1—O1—Zn1	123.6 (2)	N1—C11—H11B	109.5
Zn2—O1—Zn1	115.56 (11)	H11A—C11—H11B	109.5
C7—O2—Zn1	130.1 (2)	N1—C11—H11C	109.5
C7—O3—Zn2^iv^	112.9 (2)	H11A—C11—H11C	109.5
C8—O4—Zn1^v^	134.3 (3)	H11B—C11—H11C	109.5
C8—O5—Zn2^v^	127.6 (3)	N1—C12—H12A	109.5
C9—O6—Zn1	132.1 (3)	N1—C12—H12B	109.5
C9—O7—Zn2^vi^	128.2 (3)	H12A—C12—H12B	109.5
C10—O8—Zn1	126.1 (3)	N1—C12—H12C	109.5
C13—O9—Zn2	130.3 (3)	H12A—C12—H12C	109.5
C10—N1—C11	122.1 (4)	H12B—C12—H12C	109.5
C10—N1—C12	120.8 (4)	O9—C13—N2	123.6 (4)
C11—N1—C12	117.1 (3)	O9—C13—H13	118.2
C13—N2—C14	121.1 (4)	N2—C13—H13	118.2
C13—N2—C15	122.1 (4)	N2—C14—H14A	109.5
C14—N2—C15	116.7 (4)	N2—C14—H14B	109.5
O1—C1—C6	118.9 (3)	H14A—C14—H14B	109.5
O1—C1—C2	123.9 (3)	N2—C14—H14C	109.5
C6—C1—C2	117.2 (3)	H14A—C14—H14C	109.5
C3—C2—C1	119.5 (3)	H14B—C14—H14C	109.5
C3—C2—C7	116.9 (3)	N2—C15—H15A	109.5
C1—C2—C7	123.6 (3)	N2—C15—H15B	109.5
C4—C3—C2	122.3 (3)	H15A—C15—H15B	109.5
C4—C3—H3	118.9	N2—C15—H15C	109.5
C2—C3—H3	118.9	H15A—C15—H15C	109.5
C3—C4—C5	118.4 (3)	H15B—C15—H15C	109.5
			
O5^i^—Zn2—O1—C1	176.2 (3)	C6—C1—C2—C3	−0.6 (5)
O3^ii^—Zn2—O1—C1	1.2 (3)	O1—C1—C2—C7	−1.7 (6)
O7^iii^—Zn2—O1—C1	−90.2 (3)	C6—C1—C2—C7	176.7 (4)
O9—Zn2—O1—C1	83.2 (3)	C1—C2—C3—C4	0.7 (6)
O5^i^—Zn2—O1—Zn1	−0.87 (17)	C7—C2—C3—C4	−176.7 (4)
O3^ii^—Zn2—O1—Zn1	−175.84 (12)	C2—C3—C4—C5	0.0 (6)
O7^iii^—Zn2—O1—Zn1	92.75 (14)	C2—C3—C4—C8	180.0 (4)
O9—Zn2—O1—Zn1	−93.80 (14)	C3—C4—C5—C6	−0.9 (6)
O4^i^—Zn1—O1—C1	162.9 (3)	C8—C4—C5—C6	179.2 (4)
O2—Zn1—O1—C1	31.5 (3)	C4—C5—C6—C1	1.0 (6)
O6—Zn1—O1—C1	−82.5 (3)	O1—C1—C6—C5	178.2 (4)
O8—Zn1—O1—C1	63.1 (15)	C2—C1—C6—C5	−0.3 (6)
O4^i^—Zn1—O1—Zn2	−20.14 (16)	Zn2^iv^—O3—C7—O2	−22.4 (5)
O2—Zn1—O1—Zn2	−151.52 (15)	Zn2^iv^—O3—C7—C2	156.6 (3)
O6—Zn1—O1—Zn2	94.47 (16)	Zn1—O2—C7—O3	−165.0 (3)
O8—Zn1—O1—Zn2	−120.0 (14)	Zn1—O2—C7—C2	16.1 (5)
O4^i^—Zn1—O2—C7	−125.3 (3)	C3—C2—C7—O3	5.2 (5)
O6—Zn1—O2—C7	59.7 (4)	C1—C2—C7—O3	−172.1 (4)
O1—Zn1—O2—C7	−29.2 (3)	C3—C2—C7—O2	−175.8 (4)
O8—Zn1—O2—C7	153.1 (3)	C1—C2—C7—O2	6.9 (6)
O4^i^—Zn1—O6—C9	−99.1 (4)	Zn1^v^—O4—C8—O5	−36.5 (6)
O2—Zn1—O6—C9	76.7 (4)	Zn1^v^—O4—C8—C4	142.9 (3)
O1—Zn1—O6—C9	163.8 (4)	Zn2^v^—O5—C8—O4	−9.7 (6)
O8—Zn1—O6—C9	−13.7 (4)	Zn2^v^—O5—C8—C4	170.9 (3)
O4^i^—Zn1—O8—C10	−149.8 (4)	C3—C4—C8—O4	1.8 (6)
O2—Zn1—O8—C10	−18.0 (3)	C5—C4—C8—O4	−178.2 (4)
O6—Zn1—O8—C10	96.0 (4)	C3—C4—C8—O5	−178.7 (4)
O1—Zn1—O8—C10	−49.5 (16)	C5—C4—C8—O5	1.3 (6)
O5^i^—Zn2—O9—C13	−83.7 (4)	Zn2^vi^—O7—C9—O6	173.7 (3)
O1—Zn2—O9—C13	22.1 (4)	Zn1—O6—C9—O7	−172.2 (3)
O3^ii^—Zn2—O9—C13	144.4 (4)	Zn1—O8—C10—N1	−178.9 (3)
O7^iii^—Zn2—O9—C13	157.6 (7)	C11—N1—C10—O8	−2.0 (7)
Zn2—O1—C1—C6	−18.0 (5)	C12—N1—C10—O8	179.9 (4)
Zn1—O1—C1—C6	158.8 (3)	Zn2—O9—C13—N2	−164.4 (3)
Zn2—O1—C1—C2	160.4 (3)	C14—N2—C13—O9	1.1 (7)
Zn1—O1—C1—C2	−22.9 (5)	C15—N2—C13—O9	−178.8 (5)
O1—C1—C2—C3	−179.0 (3)		

Symmetry codes: (i) *x*+1, *y*, *z*; (ii) *x*+1/2, −*y*+3/2, *z*+1/2; (iii) −*x*+3/2, *y*+1/2, −*z*+3/2; (iv) *x*−1/2, −*y*+3/2, *z*−1/2; (v) *x*−1, *y*, *z*; (vi) −*x*+3/2, *y*−1/2, −*z*+3/2.
